# The emergence of long timescales and stereotyped behaviors in *C. elegans*

**DOI:** 10.1186/1471-2202-12-S1-P13

**Published:** 2011-07-18

**Authors:** Greg J Stephens, Matthew Bueno de Mesquita, William S Ryu, William Bialek

**Affiliations:** 1 Lewis-Sigler Institute for Integrative Genomics and Physics Department, Princeton University, Princeton, NJ 08544, USA; 2 Physics Department and Banting and Best Department of Medical Research, University of Toronto, Toronto, Ontario, M5S 1A7 Canada; 3 Princeton Center for Theoretical Science, Princeton University, Princeton, NJ 08544, USA

## 

Animal behaviors are often decomposable into discrete, stereotyped elements, well-separated in time. In one model, such behaviors are triggered by specific commands; in the extreme case, the discreteness of behavior is traced to the discreteness of action potentials in the individual command neurons. Here, we use the crawling behavior of the nematode *C. elegans* to demonstrate the opposite view, in which discreteness, stereotypy and long timescales emerge from the collective dynamics of the behavior itself. In previous work [1], we found that as *C. elegans* crawls, its body moves through a ``shape space'' in which four dimensions capture ~95% of the variance in body shape. Here we show that stochastic dynamics within this shape space predicts transitions between attractors corresponding to abrupt reversals in crawling direction. With no free parameters, our inferred stochastic dynamical system generates reversal time scales and stereotyped trajectories in close agreement with experimental observations. We use the stochastic dynamics to show that the noise amplitude decreases systematically with increasing time away from food, resulting in longer bouts of forward crawling and suggesting that worms can use noise to adaptive benefit.
